# Heat-related illness and dementia: a study integrating epidemiological and experimental evidence

**DOI:** 10.1186/s13195-024-01515-7

**Published:** 2024-07-03

**Authors:** Wan-Yin Kuo, Chien-Cheng Huang, Chi-An Chen, Chung-Han Ho, Ling‑Yu Tang, Hung-Jung Lin, Shih-Bin Su, Jhi-Joung Wang, Chien-Chin Hsu, Ching-Ping Chang, How-Ran Guo

**Affiliations:** 1 Department of Emergency Medicine, Chi Mei Medical Center, 901 Zhonghua Rd., Yongkang Dist, Tainan, 71004 Taiwan (R.O.C.); 2 Department of Occupational Medicine, Chi Mei Medical Center, 901 Zhonghua Rd., Yongkang Dist, Tainan, 71004 Taiwan (R.O.C.); 3 Department of Environmental and Occupational Health, College of Medicine, National Cheng Kung University, 138 Shengli Rd., North Dist, Tainan, 70428 Taiwan (R.O.C.); 4 Department of Emergency Medicine, Kaohsiung Medical University, 100 Shih-Chuan 1st Rd., Sanmin Dist, Kaohsiung, 80708 Taiwan (R.O.C.); 5 Department of Medical Research, Chi Mei Medical Center, 901 Zhonghua Rd., Yongkang Dist, Tainan, 71004 Taiwan (R.O.C.); 6 Department of Information Management, Southern Taiwan University of Science and Technology, 1 Nantai Street, Tainan, 71005 Taiwan (R.O.C.); 7 Department of Emergency Medicine, Taipei Medical University, 250 Wuxing Street, Taipei, 11031 Taiwan (R.O.C.); 8 Department of Leisure, Recreation and Tourism Management, Southern Taiwan University of Science and Technology, 1 Nantai Street, Tainan, 71005 Taiwan (R.O.C.); 9 Department of Medical Research, Chi Mei Medical Center, 73657 Liouying, Tainan, 201 Taikang, Taiwan (R.O.C.); 10 Department of Anesthesiology, Tri-Service General Hospital & National Defense Medical Center, 161 Sec. 6, Minquan East Road, Taipei, 11490 Taiwan (R.O.C.); 11 Department of Occupational and Environmental Medicine, National Cheng Kung University Hospital, 138 Shengli Road, Tainan, 70428 Taiwan (R.O.C.)

**Keywords:** Amyloid plaque, Apoptosis, Cognitive deficits, Dementia, Epidemiology, Heat related illness, Hippocampus, Neurodegeneration

## Abstract

**Background:**

Heat-related illness (HRI) is commonly considered an acute condition, and its potential long-term consequences are not well understood. We conducted a population-based cohort study and an animal experiment to evaluate whether HRI is associated with dementia later in life.

**Methods:**

The Taiwan National Health Insurance Research Database was used in the epidemiological study. We identified newly diagnosed HRI patients between 2001 and 2015, but excluded those with any pre-existing dementia, as the study cohort. Through matching by age, sex, and the index date with the study cohort, we selected individuals without HRI and without any pre-existing dementia as a comparison cohort at a 1:4 ratio. We followed each cohort member until the end of 2018 and compared the risk between the two cohorts using Cox proportional hazards regression models. In the animal experiment, we used a rat model to assess cognitive functions and the histopathological changes in the hippocampus after a heat stroke event.

**Results:**

In the epidemiological study, the study cohort consisted of 70,721 HRI patients and the comparison cohort consisted of 282,884 individuals without HRI. After adjusting for potential confounders, the HRI patients had a higher risk of dementia (adjusted hazard ratio [AHR] = 1.24; 95% confidence interval [CI]: 1.19–1.29). Patients with heat stroke had a higher risk of dementia compared with individuals without HRI (AHR = 1.26; 95% CI: 1.18–1.34). In the animal experiment, we found cognitive dysfunction evidenced by animal behavioral tests and observed remarkable neuronal damage, degeneration, apoptosis, and amyloid plaque deposition in the hippocampus after a heat stroke event.

**Conclusions:**

Our epidemiological study indicated that HRI elevated the risk of dementia. This finding was substantiated by the histopathological features observed in the hippocampus, along with the cognitive impairments detected, in the experimental heat stroke rat model.

**Supplementary Information:**

The online version contains supplementary material available at 10.1186/s13195-024-01515-7.

## Background

Concerns about heat-related illness (HRI) are escalating due to global warming. HRI occurs when the core body temperature surpasses the compensatory limits of thermoregulation and is a spectrum of diseases ranging from minor conditions such as heat cramps to life-threatening heat stroke [1–3]. Heat stroke is characterized by a core body temperature > 40 °C and central nervous system (CNS) dysfunction, which can result in multi-organ failure [1, 3].

With early diagnosis and aggressive treatment, patients with HRI can fully recover within days to weeks, and long-term consequences have once been regarded as rare. Some studies have reported increased risks of cardiovascular diseases [4–7], chronic kidney disease (CKD) [8], and psychiatric disorders [9]. Nevertheless, neurological sequelae are the most frequently reported consequences after HRI events in the literature, including cerebellar syndrome, cognitive deficits, Parkinson’s disease, central pontine myelinolysis, and cerebral venous thrombosis [10–15]. The injury to the CNS improves dramatically after cooling treatments, but the precise proportion of neurological recovery or functional impairment is not well known [16]. High rates of neurological disability following heat stroke were reported in survivors at discharge during the 1995 Chicago heat wave (22%) and the 2003 France heat wave (33%) [17, 18]. A literature review of 90 cases diagnosed as environmental heat stroke has reported that 23.3% of the survivors had convalescent or long-term neurological sequelae. Of the patients with neurological sequelae, 66.7% had motor dysfunction, 9.5% had cognitive impairment, and 19% had both [19]. Although epidemiological data have demonstrated that brain damage caused by HRI may be prolonged or permanent, most studies are case reports, case series, or observational studies with limited sample sizes. A recent study that used a large sample showed a three-fold increased risk of developing dementia [9]. While HRI is known to impact the neurological system, limited evidence exists regarding whether HRI is associated with the development of dementia later in life. Therefore, we conducted a population-based cohort study at the national level, as well as an animal experiment, to address this knowledge gap.

## Methods

### Epidemiological study

#### Data source

We analyzed the National Health Insurance Research Database (NHIRD), which comprises registration files and original claim data submitted for reimbursement from the National Health Insurance (NHI) program of Taiwan. The NHI was established in 1995 and had enrolled more than 99.9% of Taiwanese citizens [20]. The NHIRD is one of the largest administrative health care databases in the world, containing comprehensive data on inpatient and outpatient care, prescribed medicines, intervention procedures, and coded diagnoses using the International Classification of Diseases, Ninth Revision, Clinical Modification (ICD-9-CM) or the International Classification of Diseases, Tenth Revision (ICD-10). The NHIRD has been utilized in healthcare research to produce evidence supporting healthcare policy-making and clinical decisions [21].

#### Study design, participants, identification of variables, and assessment of outcomes

We identified patients aged ≥ 20 years with a new diagnosis of HRI between 2001 and 2015, determined by the ICD-9-CM code of 992 or ICD-10 code of T67, for hospitalization, emergency department care, or outpatient department care as the study cohort. Among the HRI patients, we further divided them into those with heat stroke (ICD-9-CM: 992.0 or ICD-10: T67.0) and those with other HRI (ICD-9-CM: 992.1–992.9 or ICD-10: T67.1–T67.9; heat syncope, heat cramps, heat exhaustion, heat fatigue, heat edema, or other unspecified effects of heat exposure). We used the date when the HRI was first diagnosed as the index date and excluded patients with a prior diagnosis of dementia before the date. Among individuals without HRI and without previous diagnosis of dementia, we selected a comparison cohort through matching by age, sex, and the index date with the study cohort at a 1:4 ratio.

**Fig. 1:**
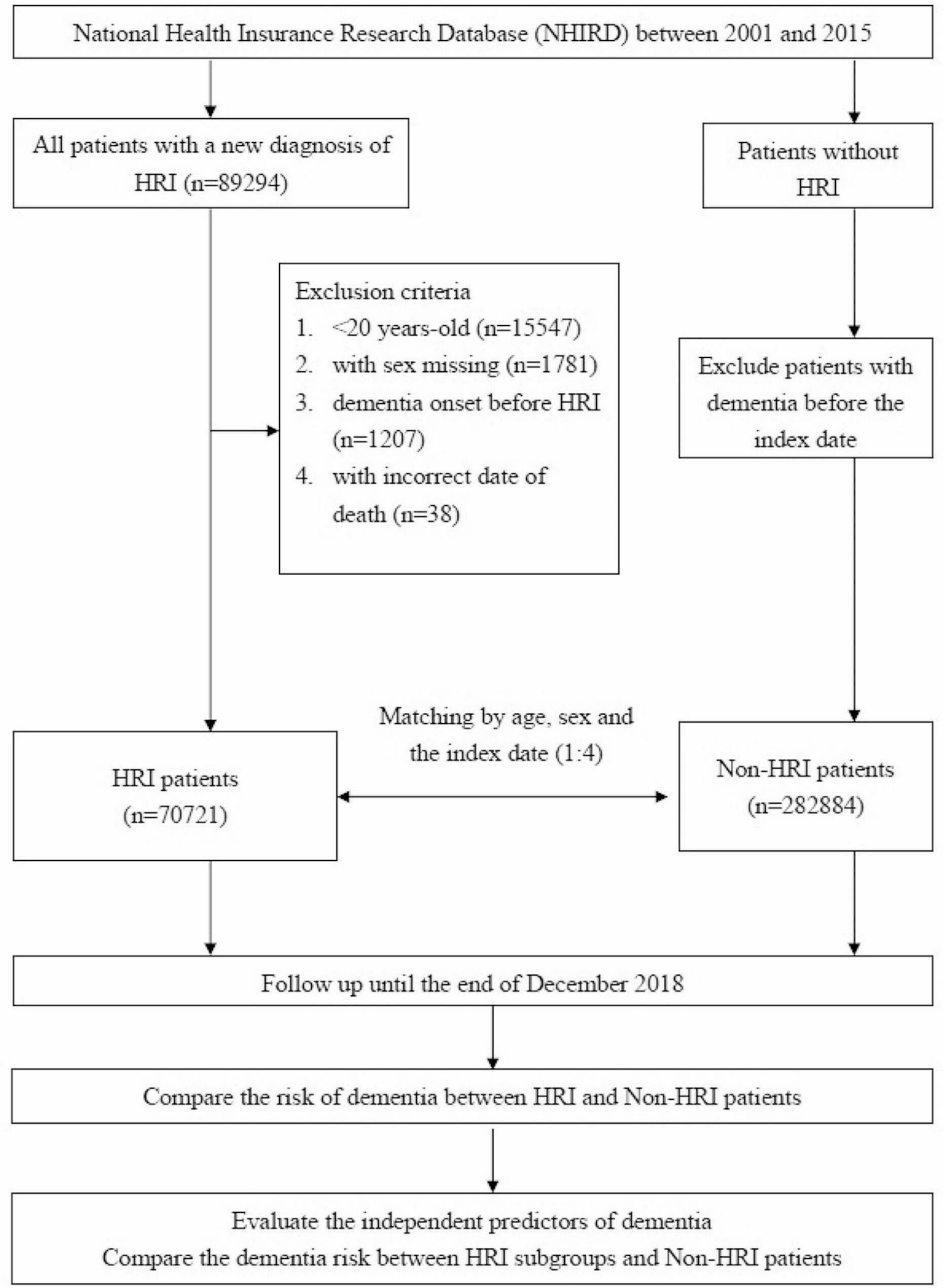
The flowchart of this study. HRI, heat-related illness

We defined patients as having dementia if they had a diagnosis code of ICD-9-CM 290.0–290.4, 294.0, 294.1, 294.8, 331.0–331.2, 331.7, or 331.82; or ICD-10 code F01–F04, F061, F068, G30, G310–G312, or G318. The definition has been used for identifying dementia in other studies [22]. To ensure the accuracy of diagnosis, we included patients who had been coded once on hospital claims or at least three times on ambulatory care claims because the diagnoses on ambulatory care claims could be just tentative diagnoses [22]. Covariates included age, sex, medical comorbidities, and monthly income. We further classified all the HRI patients into three age groups: 20–39, 40–64, and ≥ 65 years according to the Taiwanese government’s definitions of adulthood, eligibility for a comprehensive health checkup covered by the NHI, and elders [23]. We categorized monthly income into three subgroups: <20,000, 20,000-3999, and ≥ 40,000 New Taiwan Dollars (NTD). We studied medical comorbidities including hypertension, diabetes, hyperlipidemia, cardiovascular disease, chronic obstructive pulmonary disease (COPD), cerebrovascular disease, renal disease, mental disorder, Parkinson’s disease, alcoholism, and head injury. ICD-9-CM codes and ICD-10 codes for medical comorbidities were shown in the Supplementary Table 1. These 11 comorbidities were determined as being diagnosed if the patient had been coded at least three times on ambulatory care claims or once on hospital claims before a diagnosis of HRI. Each cohort member was followed until the diagnosis of the outcome under study, the end of 2018, or death, whichever came first. Subsequently, we compared the risk of dementia between the two cohorts (Fig. 1).

### Animal experiment

#### Animals

For this study, we obtained Sprague–Dawley rats from the colonies of BioLASCo Taiwan CO., Ltd. (Taipei, Taiwan). We used male rats to avoid interference of menstrual cycles. The rats were 7 weeks old and weighed between 240 and 255 g. They were housed in groups of four per cage in an environmental chamber maintained at a temperature of 24 °C and 50% relative humidity (RH). The rats were identified by a number printed on the tail base. Lighting was automatically controlled from 08:00 a.m. to 08:00 p.m. The rats had free access to standard laboratory chow and water.

#### Rat model for exertional heat stroke (EHS)

We followed the protocol established in the previous study for familiarization and induction of EHS [24]. The 24 rats were randomly divided into two groups using computer-generated randomization: an EHS group (*n* = 18) and a normothermia control (NC) group (*n* = 6). The EHS group was exposed to a high environmental temperature of 36 ± 1 °C and an RH of 50%±2%, while the NC group was maintained at a room temperature of 26 ± 1 °C and an RH of 50%±2%. The EHS group was further divided into three subgroups: EHS-onset, Day3 post-EHS, and Day14 post-EHS, with six rats in each group. EHS was triggered by progressively increasing the initial treadmill speed by 1 m/min every 2 min. until the rat appeared to be unable to run. The exhaustion was operationally defined as the third time a rat could not maintain the speed of the treadmill belt and remained on the electric shock grid for 2 s. We recorded the time to exhaustion as well as the time-dependent and velocity-dependent colonic temperature changes using a customized EHS module. Following the confirmation of exhaustion, rats in the EHS-onset group were removed from the treadmill and allowed to recover for 2 h at room temperature. After recovery, they underwent neurobehavioral testing before being euthanized with a Zoletil overdose (100 mg/kg body weight, intraperitoneally) for further brain histological examination. Rats in the Day 3 post-EHS and Day 14 post-EHS groups recovered for either 3 or 14 days, respectively, after the onset of EHS. Subsequently, these groups also underwent neurobehavioral testing and were euthanized using a Zoletil overdose and obtained brain tissue for histological examination. Rats in the NC group were kept at room temperature of 26 ± 1 °C with 50%± 2% RH without exercise. These rats maintained their levels of brightness, alertness, and responsiveness throughout the entire experiment.

#### Histopathological studies

We performed histological examinations of rats at EHS-onset, Day3 post-EHS, and Day14 post-EHS. Brains were formalin-fixed and embedded into paraffin blocks. Serial sections (4 μm) through the hippocampus (ranging from − 2.8 mm to − 4.3 mm posterior to bregma) were stained with hematoxylin and eosin (HE) for microscopy. Unaware of the experimental conditions, inspectors assessed neuronal damage in each section using two grading systems: a lesion score from 0 (no pathological changes) to 4 (lesions involving 75–100%) [25] and a morphology score from 0 (normal) to 3 (severe damage) [26]. We multiplied the two scores as the damage score [27].

#### Aβ plaque stain

The sections were stained for amyloid plaques using Thioflavin-S. After deparaffinization and hydration, they were incubated in 0.25% potassium permanganate solution for 20 min., rinsed in distilled water, and incubated in a bleaching solution (2% oxalic acid and 1% potassium metabisulfite) for 2 min. After rinsing in distilled water, the sections were transferred to a blocking solution (1% sodium hydroxide and 0.9% hydrogen peroxide) for 20 min. They were then incubated for 5 s in 0.25% acidic acid, washed in distilled water, and stained with 0.0125% Thioflavin-S in 50% ethanol for 5 min. Finally, the sections were washed with 50% ethanol, placed in distilled water, and covered with a glass cover using a mounting solution [28].

#### Triple immunofluorescence staining

The process began with the identification of NeuN + cells using the specific Neu-N antibody (1:200, MAB377, Merck Millipore, Billerica, MA, USA). This was followed by incubation with Alexa Fluor 568–conjugated goat anti-mouse IgG (#A11004, Invitrogen), then excitation at 578 nm and observation through 603 nm emission. Counterstaining was performed with 4’, 6-diamidino-2 phenylindole (DAPI, 1:50,000, excitation/emission wavelengths: 358/461; #62,247, Thermo Fisher Scientific Inc., MA, USA) for nuclear identification.

For the detection of degenerative neurons, brain tissue slides were immersed sequentially in solutions of 5% sodium hydroxide and 100% ethanol for 5 min., 70% ethanol for 2 min., distilled water for 2 min., and 0.06% potassium permanganate solution for 10 min. after secondary antibody incubation. Slides were then rinsed twice in distilled water for 2 min. and incubated in a 0.0004% Fluoro-Jade B solution (#AG310, Millipore, MA, USA), created by adding 4 ml of 0.01% stock solution to 96 ml of 0.1% acetic acid. Following 20 min. incubation in the Fluoro-Jade B staining solution, the slides were thoroughly washed, dehydrated, and coverslipped [27].

For the detection of neuronal apoptosis, brain slides underwent treatment with proteinase K (20 µg/ml) for 15 min. at room temperature prior to primary antibody incubation. Subsequently, an equilibration buffer was applied for 10 s, and the slides were immersed in a working strength terminal deoxynucleotidyl transferase (TdT) enzyme solution at 37 °C for 1 h. The reaction was then terminated using a stop/wash buffer for 10 min. The slides were incubated in working strength anti-digoxigenin conjugate at room temperature in the dark for 30 min. to visualize DNA fragments, using the terminal deoxyribonucleotide transferase-mediated dUTP nick end labeling (TUNEL) assay kit (excitation/emission wavelengths: 480/520 nm; #630,108, Takara Bio Inc., CA, USA). TUNEL-positive neurons with condensed nuclei were identified as deceased or apoptotic neurons.

A final wash was conducted with PBS, and the slides were mounted using glycerol gelatine mounting medium (#GG1-15 ML, Sigma-Aldrich, St. Louis, MO, USA) and examined using an upright fluorescence microscope (Carl Zeiss Microscopy GmbH, Jena, Germany). Images were captured with a digital camera linked to a computer running Axioscope Version 4 (Carl Zeiss), and a pathologist quantified the average percentage of Fluoro-Jade + NeuN/DAPI and TUNEL + NeuN/DAPI triple-labeled cells in six fields per section in the hippocampus (×400 magnification).

#### Animal behavioral tests: radial maze assay (reference memory and working memory test)

The maze consisted of eight arms extending radially from a central area. The arms were positioned 50 cm above the floor in a dimly lit room with visual cues. At the end of each arm, there was a single food pellet. Before the training, rats were placed to explore the maze for 5 min. and consume food freely. The animals were trained for 3 days to run to the end of the arms and consume the baited food. The training trial continued until all the 4 baits had been used up or until 5 min. had passed. After adaptation, all the rats were trained with one trial per day for 7 consecutive days. Each rat was assessed for working and reference memory, in which the same four arms were baited. When the rats made 7 or 8 correct choices and less than one error in three sessions, they were considered for the experiment.

Each rat was evaluated at EHS-onset, Day3 post-EHS, Day7 post-EHS, and Day14 post-EHS (*n* = 6) by recording the number of working memory errors (entering an arm containing food that was previously entered) and reference memory errors (entering an arm that was never baited). Reference memory is a long-term process for information that remains constant over repeated trials, and working memory is a short-time memory process in which the information to be remembered changes in every trial [29].

#### Animal behavioral tests: passive avoidance test

In the passive avoidance test which was previously described [30, 31], a single, inescapable scrambled electric shock was administered for 3 s once the rat entered the dark chamber. The time taken by the rat to enter the dark compartment from the light compartment was recorded as the testing latency. Each rat was evaluated on EHS onset and Days 3, 7, and 14 post-EHS.

#### Animal behavioral tests: rotarod assay

A rotarod treadmill (ENV576; Med Associates, St Albans, VT, USA) was used to evaluate the rats’ motor coordination before and during the post-EHS recovery phase. The settings of the rotarod were adjusted to gradually increase the speed from 4 to 30 rpm over a span of 5 min., and the most extended duration each rat remained on the treadmill was recorded, with a maximum allowable time of 5 min. The resulting data for each animal was expressed as a percentage of the baseline values.

### Statistical analysis

In the epidemiological study, we evaluated differences between the two cohorts using Student’s t-test for continuous variables and Pearson’s chi-square test for categorical variables. Cox proportional hazards regression models were used to compare the risk of dementia. We performed multivariable analyses to adjust for potential confounders. We also conducted survival analysis using the Kaplan-Meier method and log-rank test. All statistical analyses were performed using Statistical Analysis Software (SAS) Version 9.4 (SAS Institute, Cary, NC, USA) at the significance level of 0.05 (two-tailed).

In the animal experiment, the person charged with functional outcome measurements was the only one blinded to experiments among those working on animals (single-blind). She used animal codes to recognize individual rats and to report repeated measurements on data collection forms. Data were presented as the mean ± standard deviation. For analysis of behavior parameters (radial maze, passive avoidance, and rotarod), we performed one-way ANOVA followed by Tukey’s post hoc test. The parameters with a non-normal distribution–such as histological scores–were evaluated using the Kruskal-Wallis test with Dunn’s post hoc test. Statistical analysis was performed using GraphPad Prism software (Version 7.01 for Windows, GraphPad Software, San Diego, CA, USA), and the significance level was set at 0.05 (two-tailed).

## Results

### Descriptive characteristics of the study population

The study cohort consisted of 70,721 HRI patients, and the comparison cohort consisted of 282,884 individuals. In both cohorts, the mean age was 47.5 ± 17.9 years, and 55.8% of the members were male. The study cohort had higher prevalence of hypertension, COPD, cerebrovascular disease, cardiovascular disease, renal disease, mental disorder, alcoholism, and head injury compared with the comparison cohort (Table 1).

**Table 1:** Demographic characteristics of the study population

	HRI cohort		Comparison cohort		
	(*N* = 70,721)		(*N* = 282,884)		
	N	%	N	%	***p*** **value**
Male	39,442	55.8	157,768	55.8	> 0.95
Age (years)					
Mean ± Standard Deviation	47.5 ± 17.9		47.5 ± 17.9		> 0.95
Groups					> 0.95
20–39	25,514	36.1	102,056	36.1	
40–64	29,704	42.0	118,816	42.0	
≥ 65	15,503	21.9	62,012	21.9	
Comorbidities					
Hypertension	14,969	21.2	53,177	18.8	< 0.001
Diabetes	6345	9.0	24,910	8.8	0.164
Hyperlipidemia	6163	8.7	24,100	8.5	0.097
COPD	2241	3.2	6906	2.4	< 0.001
Cerebrovascular disease	2413	3.4	8639	3.1	< 0.001
Cardiovascular disease	6751	9.6	22,905	8.1	< 0.001
Renal disease	3641	5.2	9530	3.4	< 0.001
Parkinson’s disease	291	0.4	1196	0.4	0.678
Mental disorder	7925	11.2	20,091	7.1	< 0.001
Alcoholism	784	1.1	962	0.3	< 0.001
Head injury	608	0.9	1483	0.5	< 0.001
Monthly income ^*^					< 0.001
NTD < 20,000	44,495	65.8	154,048	56.8	
NTD 20,000–39,999	15,499	22.9	67,994	25.1	
NTD ≥ 40,000	7595	11.2	49,369	18.2	

HRI, heat-related illness; COPD, chronic obstructive pulmonary disease; NTD, New Taiwan Dollars

^*^Not all the patients had identified information of the monthly income

### **Risk of dementia after HRI**

**Fig. 2:**
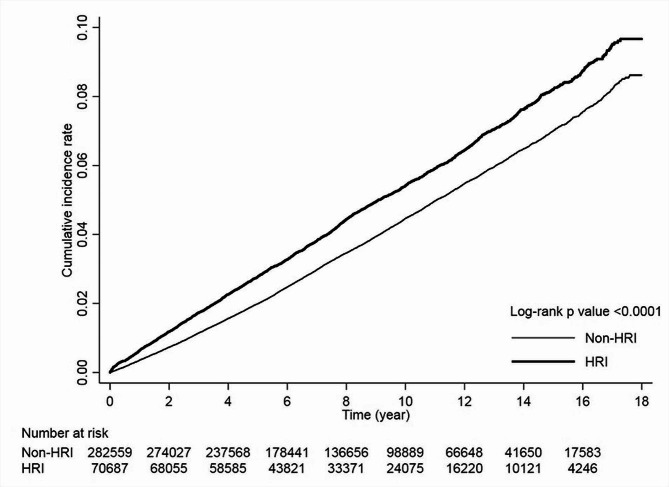
Cumulative incidence rates of dementia in the HRI patients and the non-HRI patients. HRI, heat-related illness

After adjusting for hypertension, COPD, cerebrovascular disease, cardiovascular disease, renal disease, mental disease, alcoholism and head injury, the HRI patients had a higher risk of dementia (adjusted hazard ratio [AHR] = 1.24; 95% confidence interval [CI]: 1.19–1.29) (Table 2). Furthermore, hypertension, COPD, cerebrovascular disease, cardiovascular disease, renal disease, mental disorder, alcoholism and head injury were also identified as independent predictors for dementia (Table 2). Stratified analyses showed that HRI was associated with an increased risk of dementia in all subgroups of sex and monthly income, as well as those with underlying comorbidities of hypertension, diabetes, hyperlipidemia, cerebrovascular disease, cardiovascular disease, and renal disease. The interaction effect between HRI and sex was statistically significant, with the effect of HRI on dementia risk being attenuated for females compared to males (beta= -0.1745, *p* < 0.001 for interaction effect between HRI and sex). In age subgroups, increased risks of dementia were found in HRI patients in the age groups of 40–64 and ≥ 65 (Table 3). Compared with non-HRI patients, heat stroke patients had a higher risk of dementia (AHR = 1.26; 95% CI:1.18–1.34) (Table 4). The Kaplan-Meier curves for cumulative risk showed that the study cohort had a higher risk of dementia than the comparison cohort (*p* < 0.001 for the log-rank test) (Fig. 2).

**Table 2:** Hazard ratios (HRs) of dementia obtained using Cox proportional hazards regression analysis

	Crude HR (95% CI)	*p* value	Adjusted HR^*^ (95% CI)	*p* value	Adjusted HR^†^ (95% CI)	*p* value
HRI	1.29 (1.24–1.34)	< 0.001	1.24 (1.19–1.29)	< 0.001	1.24 (1.19–1.29)	< 0.001
Comorbidities						
Hypertension	1.22 (1.18–1.28)	< 0.001	1.09 (1.04–1.14)	0.002	1.09 (1.04–1.14)	0.001
COPD	1.25 (1.16–1.35)	< 0.001	1.14 (1.05–1.23)	0.002	1.12 (1.04–1.22)	0.004
Cerebrovascular disease	1.72 (1.61–1.83)	< 0.001	1.56 (1.46–1.67)	< 0.001	1.54 (1.44–1.65)	< 0.001
Cardiovascular disease	1.22 (1.17–1.28)	< 0.001	1.08 (1.03–1.13)	0.003	1.07 (1.02–1.13)	0.009
Renal disease	1.41 (1.31–1.53)	< 0.001	1.31 (1.21–1.42)	< 0.001	1.31 (1.21–1.42)	< 0.001
Mental disorder	1.89 (1.79–1.99)	< 0.001	1.74 (1.65–1.84)	< 0.001	1.74 (1.64–1.84)	< 0.001
Alcoholism	4.04 (2.93–5.56)	< 0.001	2.80 (2.02–3.89)	< 0.001	2.72 (1.94–3.82)	< 0.001
Head injury	2.28 (1.90–2.73)	< 0.001	1.93 (1.60–2.33)	< 0.001	1.92 (1.59–2.32)	< 0.001
Monthly income^‡^						
NTD < 20,000	1 (ref)				1 (ref)	
NTD 20,000–39,999	0.94 (0.89–1.00)	0.046			0.95 (0.90–1.01)	0.123
NTD ≥ 40,000	0.92 (0.87–0.98)	0.010			0.96 (0.90–1.02)	0.139

HRI, heat-related illness; 95% CI, 95% confidence interval; COPD, chronic obstructive pulmonary disease; NTD, New Taiwan Dollars

^*^Adjusted for hypertension, COPD, cerebrovascular disease, cardiovascular disease, renal disease, mental disease, alcoholism and head injury

^†^Adjusted for hypertension, COPD, cerebrovascular disease, cardiovascular disease, renal disease, mental disease, alcoholism, head injury and monthly income

^‡^Not all the patients had identified information of the monthly income

**Table 3:** Hazard ratios (HRs) of dementia obtained using Cox proportional hazard regression analysis, stratified by sex, age, or comorbidities

	Crude HR (95% CI)	*p* value	Adjusted HR^*^ (95% CI)	*p* value
Overall analysis	1.29 (1.24–1.34)	< 0.001	1.24 (1.19–1.29)	< 0.001
Stratified analyses				
Sex				
Male	1.35 (1.28–1.43)	< 0.001	1.30 (1.23–1.37)	< 0.001
Female	1.15 (1.09–1.21)	< 0.001	1.10 (1.04–1.16)	0.001
Age group				
20–39	1.77 (1.34–2.34)	< 0.001	1.31 (0.98–1.75)	0.064
40–64	1.29 (1.18–1.41)	< 0.001	1.11 (1.01–1.21)	0.023
≥ 65	1.24 (1.19–1.30)	< 0.001	1.20 (1.15–1.25)	< 0.001
Comorbidities				
Hypertension	1.12 (1.06–1.18)	0.001	1.18 (1.11–1.24)	< 0.001
Diabetes	1.16 (1.06–1.26)	0.001	1.20 (1.10–1.30)	< 0.001
Hyperlipidemia	1.19 (1.07–1.31)	0.001	1.24 (1.12–1.37)	< 0.001
COPD	1.10 (0.96–1.25)	0.170	1.12 (0.98–1.28)	0.097
Cerebrovascular disease	1.15 (1.03–1.28)	0.014	1.22 (1.09–1.36)	< 0.001
Cardiovascular disease	1.10 (1.02–1.19)	0.015	1.18 (1.09–1.27)	< 0.001
Renal disease	0.98 (0.85–1.12)	0.714	1.21 (1.05–1.38)	0.007
Parkinson’s disease	1.22 (0.95–1.55)	0.119	1.22 (0.96–1.56)	0.111
Mental disorder	0.97 (0.89–1.05)	0.424	1.05 (0.96–1.14)	0.276
Alcoholism	0.72 (0.47–1.13)	0.151	0.78 (0.50–1.23)	0.288
Head injury	1.05 (0.78–1.40)	0.768	1.14 (0.84–1.53)	0.396
Monthly income^†^				
NTD < 20,000	1.17 (1.12–1.23)	< 0.001	1.15 (1.10–1.20)	< 0.001
NTD 20,000–39,999	1.20 (1.08–1.34)	0.001	1.14 (1.02–1.27)	0.022
NTD ≥ 40,000	1.43 (1.26–1.61)	< 0.001	1.43 (1.26–1.61)	< 0.001

HRI, heat-related illness; 95% CI, 95% confidence interval; COPD, chronic obstructive pulmonary disease; NTD, New Taiwan Dollars

^*^Adjusted for hypertension, COPD, cerebrovascular disease, cardiovascular disease, renal disease, mental disease, alcoholism and head injury

^†^Not all the patients had identified information of the monthly income

*P* < 0.001 for interaction between HRI and sex

*P* < 0.001 for interaction between HRI and age group

**Table 4:** Risk of dementia associated with different subgroups of heat-related illness (HRI).

	Number	Dementia events	Crude HR (95%CI)	*p* value	Adjusted HR^*^ (95%CI)	*p* value
All HRI	70,721	3316	1.29 (1.24–1.34)	< 0.001	1.24 (1.19–1.29)	< 0.001
Heat stroke	27,004	1409	1.31 (1.23–1.39)	< 0.001	1.26 (1.18–1.34)	< 0.001
Other HRI	43,717	1907	1.28 (1.21–1.35)	< 0.001	1.22 (1.16–1.29)	< 0.001

HR, hazard ratio; 95% CI, 95% confidence interval

^*^Adjusted for hypertension, COPD, cerebrovascular disease, cardiovascular disease, renal disease, mental disease, alcoholism and head injury

The mean follow-up time of the HRI cohort and the comparison cohort was 8.30 ± 4.37 years and 8.43 ± 4.35 years (*p* < 0.01), respectively. The median time between the onset of HRI and dementia was also shorter in the HRI cohort; 4.40 years (interquartile range: 2.0–7.8 years) vs. 5.23 years (2.62–8.62 years), *p* < 0.01.

### Animal behavioral tests after a heat stroke event

**Fig. 3:**
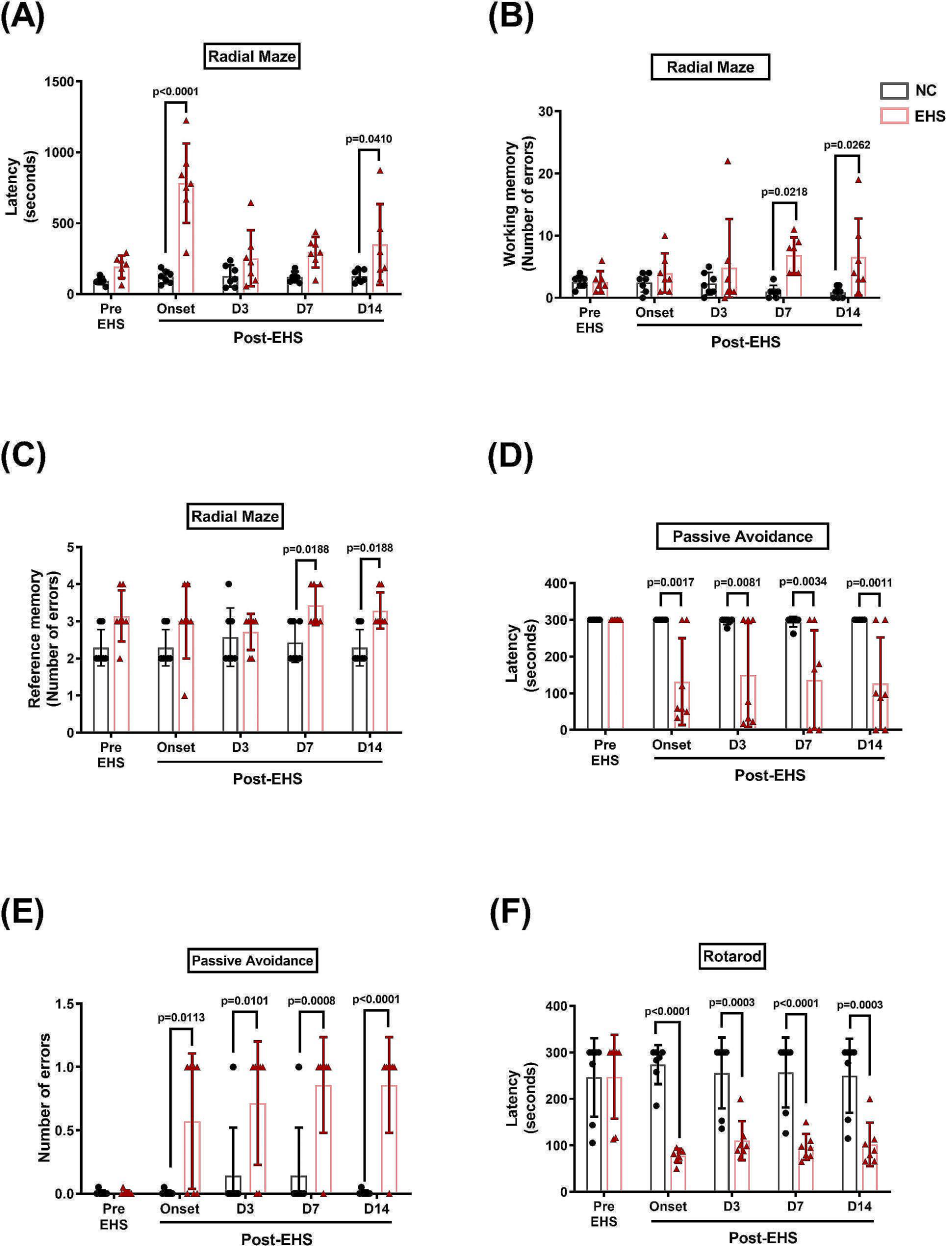
EHS causes neurobehavioral disorders. The behavioral test battery included two tests for learning and memory (radial maze and passive avoidance) and one motor test (rotarod). These tests were performed before the stress (pre-EHS) and at the onset (Onset), as well as on day 3 (D3), day 7 (D7), and day 14 (D14) of the post-EHS period for both the experimental (EHS) and normal control (NC) groups of rats. Compared to the NC group, the (**A**) retention time (latency period, seconds), (**B**) working memory errors, and (**C**) reference memory errors on the radial-arm maze test were significantly increased in EHS groups. On the passive avoidance test, the retention latency (**D**) was significantly shorter, and the number of errors (**E**) was significantly higher in EHS rats than in NC animals from Onset to D14. The EHS groups had significantly decreased (**F**) the latency (s) in the rotarod test compared to the NC group. Data were presented as mean ± standard deviation

To assess the impact of EHS on the learning and memory capabilities, as well as motor coordination, of rats, we conducted a series of tests, including the radial maze, passive avoidance, and rotarod. In the radial maze assay, the EHS-onset and Day14 post-EHS groups had longer latencies than the NC group (Fig. 3A). The working/reference memory errors were also increased in the Day14 post-EHS group compared with the NC group (Fig. 3B and C).

In the passive avoidance test, the retention latencies in the EHS-onset, Day3 post-EHS, and Day14 post-EHS groups were shorter than that in the NC group (Fig. 3D). In addition, the number of errors increased in the EHS-onset, Day3 post-EHS, and Day14 post-EHS groups compared to the NC group (Fig. 3E).

EHS rats also displayed impaired motor function in the rotarod test compared with the NC rats throughout the entire test period, starting from the onset of EHS (Fig. 3F).

### Histopathological findings and results of Aβ plaque stain

**Fig. 4:**
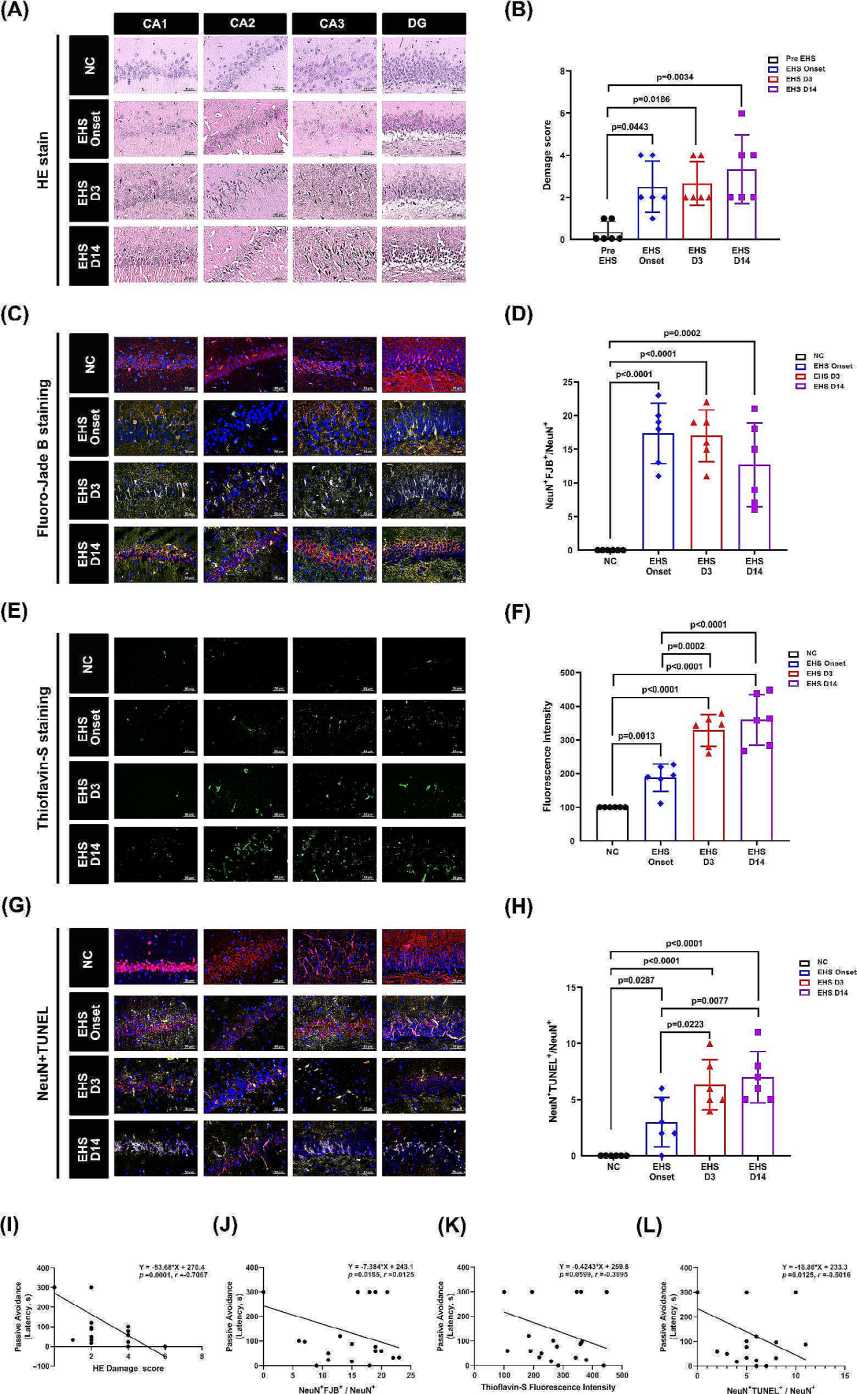
Histological analysis in NC rats and EHS rats and correlation analysis between cognitive test and histological effects. The histology of the hippocampal regions CA1, CA2, CA3, and the dentate gyrus in NC and EHS rats was examined using HE staining (**A** and **B**), NeuN + Fluoro-Jade B staining (**C** and **D**), thioflavin-S staining (**E** and **F**), and NeuN + TUNEL staining (**G** and **H**). A correlation analysis was also conducted between the passive avoidance test and various staining methods, including HE (I), NeuN + Fluoro-Jade B (**J**), thioflavin-S (**K**), and NeuN + TUNEL (**L**). A total of six animals were used for each experimental group. Data presented as mean ± standard deviation

After EHS induction, the rats’ hippocampus showed remarkable damage and multiple histopathological features, including structural disorganization, edema, pyknotic cells, and vacuolization (Fig. 4A). These phenomena were sustained to Day14 post-EHS. The damage scores of the hippocampus in the EHS-onset, Day3 post-EHS, and Day14 post-EHS groups were higher than that in the NC group (Fig. 4B). Rats with EHS exhibited neuronal degeneration (evidenced by NeuN with Fluoro-Jade B [FJB] staining; Fig. 4C and D), Aβ accumulation (evidenced by thioflavin-S [Thio-S] staining; Fig. 4E and F), and apoptosis (evidenced by NeuN and TUNEL staining; Fig. 4G and H). In addition, the Day14 post-EHS group showed the highest level of Aβ plaques accumulation in the hippocampus (Fig. 4F). Additional analyses were conducted to examine correlations between the passive avoidance test and the histological alterations under various stains. Notably, results from the passive avoidance test showed correlations with the HE, FJB, and TUNEL staining results (Fig. 4).

## Discussion

This study comprised a population-based cohort study and an animal experiment that investigated the risk of dementia after HRI events. The epidemiological study revealed that HRI was associated with an increased risk of dementia after adjusting for potential confounders. Observations in the animal experiment supported findings in the epidemiological study, including cognitive dysfunction evidenced by animal behavioral tests, as well as remarkable damages and plaque deposition in the hippocampus. In addition to HRI, other independent predictors for dementia identified in this study included hypertension, COPD, cerebrovascular disease, cardiovascular disease, renal disease, mental disorder, alcoholism, and head injury. HRI was associated with an increased risk of dementia, particularly in males and those with underlying comorbidities of hypertension, diabetes, hyperlipidemia, cerebrovascular disease, cardiovascular disease, and renal disease.

Chronic sequelae on the CNS caused by HRI have been reported in epidemiological studies. However, most of the studies assessed the functional status or neurological manifestations instead of diagnosing dementia [17–19]. In a recent study by Li et al. that evaluated the association between HRI and psychiatric disorders, HRI patients were found to have a higher risk of developing dementia (AHR = 3.18, 95% CI: 2.91–3.47, *p* < 0.001) [9], which is compatible to the findings in our study. While the two studies had different aims, applied different sampling strategies, utilized different variable measurements, and had different follow-up periods, both studies found that HRI was associated with an increased risk of dementia, highlighting the significance of this issue. In addition, we found that hypertension, COPD, cerebrovascular disease, cardiovascular disease, renal disease, mental disorder, alcoholism, and head injury were also independent predictors for dementia, which was compatible with previous studies [32–36].

When exposed to intense heat loads caused by exogenous heat, endogenous heat, or both, the human body’s thermoregulatory ability may be strained or overwhelmed, leading to HRI. Heat stroke, the most severe form of HRI, is categorized into classic and exertional. Both types can lead to a similar cascade of physiological abnormalities arising from the body’s incapacity to dissipate excessive heat [37]. When assessing learning and memory, the hippocampus is of utmost importance. It is well known that the hippocampus plays a critical role in learning and memory, and stress can impair hippocampus-dependent memory [38]. Our study revealed neuronal damage, neuronal degeneration, apoptosis, and plaque accumulation in the hippocampus, along with a decline in the rats’ ability in learning and memory, which is consistent with existing knowledge and supports our rationale. Furthermore, on Day 14 post-EHS, the hippocampus exhibited the highest Aβ plaque accumulation, suggesting irreversible adverse effects following a heat stroke event. This implies that the effects may be prolonged and even potentially permanent.

The mechanism of the development of dementia caused by heat stress remains unclear and is presumed to be multifactorial. Heat stress may result in neuronal damage via cellular, local, and systemic effects [39]. Heat stress can cause immediate motor and cognitive impairments, affecting attention, memory, and executive function. Previous studies have found a decline in cognitive performance during exposure to high temperatures [40–42]. The effects of chronic heat exposure on cognitive function are an ongoing area of research. The interplay between heat and human physiology is complex, and many variables can influence outcomes, such as individual differences, intensity, duration of exposure, hydration status, and more [43]. A study conducted on workers exposed to high heat conditions found that long-term heat stress impaired cognitive function and attention [44]. Chronic heat exposure can cause protein misfolding and aggregation [45, 46], neuroinflammation [47], and oxidative stress [48, 49], which might play a role in the pathogenesis of neurodegenerative diseases such as Alzheimer’s disease and Parkinson’s disease. A study of mice exposed to heat showed that heat stress might impair cognitive function by leading to neuroinflammation, neurodegeneration, and defective neurogenesis in the hippocampus [38]. A previous animal study also showed that hyperthermia had the potential to induce a molecular phenotype resembling Alzheimer’s disease, with upregulation of Aβ expression and phosphorylated Tau deposition [50]. After a review of the literature, Bongioanni et al. proposed heat-affected pathways in brain cells that might cause the onset of neurodegenerative diseases. They suggested that heat exposure could lead to neurodegeneration by disrupting mitochondrial function, impairing the biochemical processes amending protein misfolding, and increasing oxidative stress, excitotoxicity, and neuroinflammation. These factors could contribute to the accumulation of misfolded proteins and their aggregation in neurons under unfavorable conditions [51].

In the study, we observed increased risks of dementia after HRI in both young and old age groups. Young-onset dementia refers to any dementia where symptom onset occurs at less than 65 years of age and comprises a heterogeneous range of dementias including primary dementias such as Alzheimer disease, frontotemporal and vascular dementias, genetic/familial dementias, metabolic disorders, and secondary dementias [52]. Additionally, the consideration of environmental exposures and lifestyle factors as potential risk factors for dementia has considerably increased in recent years [53]. In our study, we included all individuals with a diagnosis of dementia, regardless of age, to comprehensively assess the association between HRI and dementia across different age groups to include both young-onset dementia and elderly dementia. However, the exact mechanism and causes of the risk of dementia observed in both young and old age groups in our study require further investigation for clarification.

In the study, we observed that male patients had a higher risk of dementia than female patients when exposing to HRI. Previous studies have revealed that sex steroid hormones, lifestyle, ethnicity, and genetic polymorphisms of sex-related genes might complicate the association between sex and dementia. There could be several reasons why male patients may have a higher risk of dementia under the context of HRI. Hormonal differences between males and females may play a role in modulating the response to heat stress and its impact on cognitive health. For example, estrogen has been shown to have neuroprotective effects [54], potentially mitigating the detrimental effects of heat on the brain. Differences in behavioral patterns between males and females, such as occupation, outdoor activities, and adherence to heat safety guidelines, may influence the risk of heat-related cognitive impairment. Males may be more likely to engage in physically demanding activities in hot environments, increasing their risk of HRI and subsequent cognitive decline. Socioeconomic factors, access to healthcare, and environmental exposures may also contribute to sex differences in the risk of dementia following HRI. Overall, the interplay of biological, behavioral, and environmental factors likely contributes to the observed sex differences in the risk of dementia under the context of HRI. Further research is needed to elucidate the underlying mechanisms and pathways through which sex influences the relationship between heat exposure and cognitive health, with implications for targeted interventions and public health strategies.

### Strengths and limitations

The major strength of the study was the integration of epidemiological and experimental evidence. In the epidemiological study, we utilized a large population sample to evaluate the risk of dementia following HRI, which distinguished our study from previous ones. Additionally, we conducted an animal experiment that included behavioral tests and histopathological findings to support the findings of the epidemiological study. However, our study still had some limitations. Firstly, the NHIRD did not provide information on the etiology of HRI, family history of dementia, smoking, diet, physical inactivity, and other sociodemographic factors such as education level. Therefore, we could not investigate these factors in our study. Secondly, the animal study may not accurately represent EHS in humans. Thirdly, our animal experiment evaluated rats’ behavioral tests and histopathological findings up to 14 days after EHS. Although we observed the highest level of Aβ plaque accumulation on 14 days after EHS, longer periods may be required to observe its effects in even longer terms. Fourthly, due to the extended prodromal period of dementia and the potential for delayed diagnosis [55, 56], reverse causality, where dementia onset preceded the occurrence of HRI but got diagnosed because of the occurrence of HRI, might affect the study results. However, there was no way to identify such cases in the study population or define the length of delay diagnosis in individual patients. On top of the supporting evidence from our animal experiments, to address this issue, we performed a sensitivity analysis in which patients with dementia diagnosed within the first 5 years after the HRI event were excluded. While a portion of the HRI-related cases of dementia would have been excluded and thus the associated relative risk was reduced to 1.07, HRI remained as a significant risk factor (95% CI: 1.01–1.13, *p* = 0.024) for dementia, which supported our conclusion of an association between HRI and subsequent dementia.

## Conclusions

Our epidemiological study revealed an increased risk of dementia after an HRI event, supported by the damage and plaque deposition in the hippocampus and the cognitive dysfunction observed in the animal experiment. The mechanism remains to be explored and may be multifactorial. This study not only provides a reference for the long-term consequences of HRI but also emphasizes the importance of addressing concerns of dementia in individuals who experience HRI events.

### Electronic supplementary material

Below is the link to the electronic supplementary material.

Supplementary Material 1

## Data Availability

The data that support the findings of this study are available from the corresponding author upon reasonable request.

## Abbreviations

HRI: heat-related illness
CNS: central nervous system
CKD: chronic kidney disease
NHI: National Health Insurance
NHIRD: National Health Insurance Research Database
ICD-9-CM: International Classification of Diseases, Ninth Revision, Clinical Modification
ICD-10: International Classification of Diseases, Tenth Revision
NTD: New Taiwan Dollars
COPD: chronic obstructive pulmonary disease
RH: relative humidity
EHS: exertional heat stroke
NC: normothermia control
HE: hematoxylin and eosin
TUNEL: terminal deoxynucleotidyl transferase dUTP nick end labeling
AHR: adjusted hazard ratio
FJB: Fluoro-Jade B

## References

[1] Atha WF. Heat-related illness. Emerg Med Clin North Am. 2013;31(4):1097–108. 10.1016/j.emc.2013.07.012. 10.1016/j.emc.2013.07.012 24176481

[2] Miyake Y (2013). Pathophysiology of heat illness: Thermoregulation, risk factors, and indicators of aggravation. Japan Med Assoc J.

[3] Gauer R, Meyers BK (2019). Heat-related illnesses. Am Fam Physician.

[4] Bathini T, Thongprayoon C, Chewcharat A, Petnak T, Cheungpasitporn W, Boonpheng B, et al. Acute myocardial infarction among hospitalizations for heat stroke in the United States. J Clin Med. 2020;9(5). 10.3390/jcm9051357. 10.3390/jcm9051357 PMC7290741 32384601

[5] Nzvere FP, Tariq E, Nishanth K, Arshid A, Cancarevic I. Long-term cardiovascular diseases of heatstroke: a delayed pathophysiology putcome. Cureus. 2020;12(8):e9595. 10.7759/cureus.9595. 10.7759/cureus.9595 PMC7416985 32789098

[6] Tseng MF, Chou CL, Chung CH, Chien WC, Chen YK, Yang HC, et al. Association between heat stroke and ischemic heart disease: a national longitudinal cohort study in Taiwan. Eur J Intern Med. 2019;59:97–103. 10.1016/j.ejim.2018.09.019. 10.1016/j.ejim.2018.09.019 30297250

[7] Wang JC, Chien WC, Chu P, Chung CH, Lin CY, Tsai SH (2019). The association between heat stroke and subsequent cardiovascular diseases. PLoS ONE.

[8] Tseng MF, Chou CL, Chung CH, Chen YK, Chien WC, Feng CH, et al. Risk of chronic kidney disease in patients with heat injury: a nationwide longitudinal cohort study in Taiwan. PLoS ONE. 2020;15(7):e0235607. 10.1371/journal.pone.0235607. 10.1371/journal.pone.0235607 PMC7332078 32614909

[9] Li FL, Chien WC, Chung CH, Lai CY, Tzeng NS. Real-world evidence for the association between heat-related illness and the risk of psychiatric disorders in Taiwan. Int J Environ Res Public Health. 2022;19(13). 10.3390/ijerph19138087. 10.3390/ijerph19138087 PMC9265553 35805746

[10] Biary N, Madkour MM, Sharif H. Post-heatstroke parkinsonism and cerebellar dysfunction. Clin Neurol Neurosurg. 1995;97(1):55–. 10.1016/0303-8467(94)00065-e. 10.1016/0303-8467(94)00065-e 7788975

[11] Cao L, Wang J, Gao Y, Liang Y, Yan J, Zhang Y, et al. Magnetic resonance imaging and magnetic resonance venography features in heat stroke: a case report. BMC Neurol. 2019;19(1):133. 10.1186/s12883-019-1363-x. 10.1186/s12883-019-1363-x PMC6580543 31215399

[12] Guerrero WR, Varghese S, Savitz S, Wu TC (2013). Heat stress presenting with encephalopathy and MRI findings of diffuse cerebral injury and hemorrhage. BMC Neurol.

[13] Laxe S, Zúniga-Inestroza L, Bernabeu-Guitart M (2013). [Neurological manifestations and their functional impact in subjects who have suffered heatstroke]. Rev Neurol.

[14] McNamee T, Forsythe S, Wollmann R, Ndukwu IM. Central pontine myelinolysis in a patient with classic heat stroke. Arch Neurol. 1997;54(8):935–6. 10.1001/archneur.1997.00550200005002. 10.1001/archneur.1997.00550200005002 9267966

[15] Wang C-C, Tsai M-K, Chen I-H, Hsu Y-D, Hsueh C-W, Shiang J-CJ (2008). Neurological manifestations in a patient of heat stroke-case report and literature review. Taiwan Crit Care Med.

[16] Al Mahri S, Bouchama A, Heatstroke (2018). Handb Clin Neurol.

[17] Argaud L, Ferry T, Le QH, Marfisi A, Ciorba D, Achache P, et al. Short- and long-term outcomes of heatstroke following the 2003 heat wave in Lyon, France. Arch Intern Med. 2007;167(20):2177–83. 10.1001/archinte.167.20.ioi70147. 10.1001/archinte.167.20.ioi70147 17698677

[18] Dematte JE, O’Mara K, Buescher J, Whitney CG, Forsythe S, McNamee T, et al. Near-fatal heat stroke during the 1995 heat wave in Chicago. Ann Intern Med. 1998;129(3):173–81. 10.7326/0003-4819-129-3-199808010-00001. 10.7326/0003-4819-129-3-199808010-00001 9696724

[19] Lawton EM, Pearce H, Gabb GM. Review article: environmental heatstroke and long-term clinical neurological outcomes: a literature review of case reports and case series 2000–2016. Emerg Med Australas. 2019;31(2):163–73. 10.1111/1742-6723.12990. 10.1111/1742-6723.12990 29851280

[20] National Health Insurance Administration. Ministry of Health and Welfare, Taiwan, ROC. National Health Insurance Annual Report 2014–2015. Ministry of Health and Welfare Taipei, Taiwan, 2014.

[21] Hsieh CY, Su CC, Shao SC, Sung SF, Lin SJ, Kao Yang YH, et al. Taiwan’s National Health Insurance Research Database: past and future. Clin Epidemiol. 2019;11:349–58. 10.2147/clep.S196293. 10.2147/CLEP.S196293 PMC6509937 31118821

[22] Lai YC, Tsai KT, Ho CH, Liao JY, Tseng WZ, Petersen I, et al. Mortality rate and its determinants among people with dementia receiving home healthcare: a nationwide cohort study. Intern Emerg Med. 2023;18:2121–30. 10.1007/s11739-023-03319-3. 10.1007/s11739-023-03319-3 37253992

[23] Hsu YH, Tsai WC, Kung PT. Health examination utilization in the visually disabled population in Taiwan: a nationwide population-based study. BMC Health Serv Res. 2013;13:509. 10.1186/1472-6963-13-509. 10.1186/1472-6963-13-509 PMC3880214 24313981

[24] Lin PH, Huang KH, Tian YF, Lin CH, Chao CM, Tang LY (2021). Exertional heat stroke on fertility, erectile function, and testicular morphology in male rats. Sci Rep.

[25] Honório JE Jr., Vasconcelos GS, Rodrigues FT, Sena Filho JG, Barbosa-Filho JM, Aguiar CC, et al. Monocrotaline: histological damage and oxidant activity in brain areas of mice. Oxid Med Cell Longev. 2012;2012:697541. 10.1155/2012/697541. 10.1155/2012/697541 PMC3517861 23251721

[26] Liu X, Gu X, Li Z, Li X, Li H, Chang J (2006). Neuregulin-1/erbB-activation improves cardiac function and survival in models of ischemic, dilated, and viral cardiomyopathy. J Am Coll Cardiol.

[27] Wang YL, Chio CC, Kuo SC, Yeh CH, Ma JT, Liu WP (2022). Exercise rehabilitation and/or astragaloside attenuate amyloid-beta pathology by reversing BDNF/TrkB signaling deficits and mitochondrial dysfunction. Mol Neurobiol.

[28] Zhang Z, Obianyo O, Dall E, Du Y, Fu H, Liu X (2017). Inhibition of delta-secretase improves cognitive functions in mouse models of Alzheimer’s disease. Nat Commun.

[29] Hritcu L, Cioanca O, Hancianu M (2012). Effects of lavender oil inhalation on improving scopolamine-induced spatial memory impairment in laboratory rats. Phytomedicine.

[30] Chou W, Liu YF, Lin CH, Lin MT, Chen CC, Liu WP (2018). Exercise rehabilitation attenuates cognitive deficits in rats with traumatic brain injury by stimulating the cerebral HSP20/BDNF/TrkB signalling axis. Mol Neurobiol.

[31] Hosseini N, Alaei H, Reisi P, Radahmadi M (2013). The effect of treadmill running on passive avoidance learning in animal model of Alzheimer disease. Int J Prev Med.

[32] Livingston G, Huntley J, Sommerlad A, Ames D, Ballard C, Banerjee S (2020). Dementia prevention, intervention, and care: 2020 report of the Lancet Commission. Lancet.

[33] Elahi FM, Miller BL (2017). A clinicopathological approach to the diagnosis of dementia. Nat Rev Neurol.

[34] von Farnsworth B, Josefsson M, Wåhlin A, Nyberg L, Karalija N (2022). Association of cardiovascular risk trajectory with cognitive decline and incident dementia. Neurology.

[35] Deckers K, Camerino I, van Boxtel MP, Verhey FR, Irving K, Brayne C (2017). Dementia risk in renal dysfunction: a systematic review and meta-analysis of prospective studies. Neurology.

[36] Wang J, Li X, Lei S, Zhang D, Zhang S, Zhang H (2022). Risk of dementia or cognitive impairment in COPD patients: a meta-analysis of cohort studies. Front Aging Neurosci.

[37] Sorensen C, Hess J (2022). Treatment and prevention of heat-related illness. N Engl J Med.

[38] Lee W, Moon M, Kim HG, Lee TH, Oh MS (2015). Heat stress-induced memory impairment is associated with neuroinflammation in mice. J Neuroinflammation.

[39] Walter EJ, Carraretto M (2016). The neurological and cognitive consequences of hyperthermia. Crit Care.

[40] Gaoua N, Racinais S, Grantham J, El Massioui F (2011). Alterations in cognitive performance during passive hyperthermia are task dependent. Int J Hyperth.

[41] Hancock PA, Ross JM, Szalma JL (2007). A meta-analysis of performance response under thermal stressors. Hum Factors.

[42] Racinais S, Gaoua N, Grantham J (2008). Hyperthermia impairs short-term memory and peripheral motor drive transmission. J Physiol.

[43] Obradovich N, Migliorini R, Paulus MP, Rahwan I (2018). Empirical evidence of mental health risks posed by climate change. Proc Natl Acad Sci U S A.

[44] Varghese BM, Hansen A, Bi P, Pisaniello D (2018).

[45] Lin KC, Lin HJ, Chang CP, Lin MT (2015). Decreasing or increasing heat shock protein 72 exacerbates or attenuates heat-induced cell death, respectively, in rat hypothalamic cells. FEBS Open Bio.

[46] Chao CM, Cheng BC, Chen CY, Lin MT, Chang CP, Yang ST (2015). Proteomic analysis of hypothalamic injury in heatstroke rats. Proteomics.

[47] Chang CP, Huang WT, Cheng BC, Hsu CC, Lin MT (2007). The flavonoid baicalin protects against cerebrovascular dysfunction and brain inflammation in experimental heatstroke. Neuropharmacology.

[48] Chen SH, Lin MT, Chang CP (2013). Ischemic and oxidative damage to the hypothalamus may be responsible for heat stroke. Curr Neuropharmacol.

[49] Chang CK, Chang CP, Liu SY, Lin MT (2007). Oxidative stress and ischemic injuries in heat stroke. Prog Brain Res.

[50] Sinigaglia-Coimbra R, Cavalheiro EA, Coimbra CG (2002). Postischemic hyperthermia induces Alzheimer-like pathology in the rat brain. Acta Neuropathol.

[51] Bongioanni P, Del Carratore R, Corbianco S, Diana A, Cavallini G, Masciandaro SM (2021). Climate change and neurodegenerative diseases. Environ Res.

[52] Loi SM, Cations M, Velakoulis D (2023). Young-onset dementia diagnosis, management and care: a narrative review. Med J Aust.

[53] Bosi M, Malavolti M, Garuti C, Tondelli M, Marchesi C, Vinceti M (2022). Environmental and lifestyle risk factors for early-onset dementia: a systematic review. Acta Biomed.

[54] Dubal DB, Wise PM (2002).

[55] Draper B, Cations M, White F, Trollor J, Loy C, Brodaty H (2016). Time to diagnosis in young-onset dementia and its determinants: the INSPIRED study. Int J Geriatr Psychiatry.

[56] Davis MA, Lee KA, Harris M, Ha J, Langa KM, Bynum JPW (2022). Time to dementia diagnosis by race: a retrospective cohort study. J Am Geriatr Soc.

