# Supplementary material for: Heat-related illness and dementia: a study integrating epidemiological and experimental evidence
Source: Alzheimers Res Ther. 2024 Jul 3;16:145. doi: 10.1186/s13195-024-01515-7 (PMC11221187; doi:10.1186/s13195-024-01515-7)
Supplement: Supplementary file 1 — Supplementary Material 1 [file 13195_2024_1515_MOESM1_ESM.docx]

**Supplementary Table 1.** ICD-9-CM codes and ICD-10 codes for medical comorbidities

| Medical comorbidities | ICD-9-CM codes | ICD-10 codes |
| --- | --- | --- |
| Hypertension | 401–405 | I10–I16 |
| Diabetes | 250 | E08–E13 |
| Hyperlipidemia | 272 | E78 |
| Cardiovascular disease | 390–398, 410–429, 440–448 | I00–I02, I05–I09, I20, I26–I28, I30–I52, I70–I75, I77–I79 |
| Chronic obstructive pulmonary disease (COPD) | 490–492, 496 | J40, J410, J411, J418, J42, J430, J431, J432, J438, J439, J440, J441, J449 |
| Cerebrovascular disease | 430–438 | I60–I69 |
| Renal disease | 580–593 | N00–N20, N25–N29 |
| Mental disorder | 290–319 | F01–F99 |
| Parkinson’s disease | 332 | G20 |
| Alcoholism | 291, 303, 305.0, 357.5, 425.5, 535.3, 571.0, 571.1, 571.2, 571.3, V11.3 | F10, K70, G621, I426, K292 |
| Head injury | 800, 801, 803, 804, 850, 851, 852, 853, 854, 310.2 | S06, S020, S021, F0781, S0291 |
